# Differences in Physiological Signals Due to Age and Exercise Habits of Subjects during Cycling Exercise

**DOI:** 10.3390/s21217220

**Published:** 2021-10-29

**Authors:** Szu-Yu Lin, Chi-Wen Jao, Po-Shan Wang, Michelle Liou, Jun-Liang Wu, Hsiao Chun, Ching-Ting Tseng, Yu-Te Wu

**Affiliations:** 1 Institution of Biophotonics, National Yang Ming Chiao Tung University, Taipei 112, Taiwan; betty810720@nycu.edu.tw (S.-Y.L.); c3665810@ms24.hinet.net (C.-W.J.); b8001071@yahoo.com.tw (P.-S.W.); apply91122@gmail.com (H.C.); shps961421@gmail.com (C.-T.T.); 2 Department of Research, Shin Kong Wu Ho-Su Memorial Hospital, Taipei 111, Taiwan; 3 Department of Neurology, Municipal Gandau Hospital, Taipei 112, Taiwan; 4 Institute of Statistical Science, Academia Sinica, Taipei 115, Taiwan; mliou@stat.sinica.edu.tw; 5 Department of Health of Beitou District, Taipei City Government, Taipei 112, Taiwan; wclwclwcl@health.gov.tw; 6 Brain Research Center, National Yang Ming Chiao Tung University, Taipei 112, Taiwan

**Keywords:** exercise, EEG, EMG, ECG, brain activity, age, exercise habit

## Abstract

Numerous studies indicated the physical benefits of regular exercise, but the neurophysiological mechanisms of regular exercise in elders were less investigated. We aimed to compare changes in brain activity during exercise in elderly people and in young adults with and without regular exercise habits. A total of 36 healthy young adults (M/F:18/18) and 35 healthy elderly adults (M/F:20/15) participated in this study. According to exercise habits, each age group were classified into regular and occasional exerciser groups. ECG, EEG, and EMG signals were recorded using V-AMP with a 1-kHz sampling rate. The participants were instructed to perform three 5-min bicycle rides with different exercise loads. The EEG spectral power of elders who exercised regularly revealed the strongest positive correlation with their exercise intensity by using Pearson correlation analysis. The results demonstrate that exercise-induced significant cortical activation in the elderly participants who exercised regularly, and most of the *p*-values are less than 0.001. No significant correlation was observed between spectral power and exercise intensity in the elders who exercised occasionally. The young participants who exercised regularly had greater cardiac and neurobiological efficiency. Our results may provide a new exercise therapy reference for adult groups with different exercise habits, especially for the elders.

## 1. Introduction

Regular physical exercise is associated with health benefits and is a crucial element of preventive strategies for promoting health. During exercise, moving the body requires a substantial degree of brain activity, necessitating the activation of numerous neurons to generate, receive, and interpret repeated, rapid-fire messages from the nervous system [1]. However, the neurophysiological mechanisms underlying the effects of exercise are poorly understood and require further investigation. Cycling is a common exercise, and daily cycling can enable a large proportion of the population to meet their recommended physical activity levels [2]. Several studies have reported that cycle ergometers are suitable for measuring physiological signals emitted during exercise. Studies on cycling exercise have reported that such exercise can induce specific changes in cortical activity. These changes are measured through various methods, including electroencephalography (EEG), the aim of which is to study the modulation of brain activity associated with performing cycling tasks [3,4,5,6,7].

Hottenrott et al. reported that cortical brain activation could be measured during cycling exercise; they suggested that higher cortical brain activation is necessary to increase muscle strength at higher cadences [4]. Enders et al. recently revealed that EEG power increased significantly in the frontal cortex and parietal cortex as fatigue accumulated throughout high-intensity cycling exercise activities. Notably, they observed a broadband increase in EEG power, in contrast to other studies that investigated various exercise conditions and observed changes that were limited to the alpha and beta bands [5]. Brummer et al. localized the exercise-induced changes in brain cortical activity by using the active-EEG/low-resolution electromagnetic tomography analysis and demonstrated that motor cortex activity increased with additional exercise intensity on a cycle ergometer [6]. Although Brummer et al. used different methodologies from other, earlier research, all of the aforementioned studies have focused on the effects of exercise intensity on cortical activity in young people or athletes [7]. Few studies have examined the activity of the cerebral cortex during exercise in other segments of the population, especially in older adults. Moreover, few studies have investigated the neurobiological differences between regular and occasional exercisers during physical exercise.

Accordingly, the aim of the present study was to investigate the changes in brain activity during exercise in elderly people and young adults. Previous studies have proposed the use of heart rate as a measure of exercise intensity [8]. They have described a positive linear correlation between increasing exercise intensity and changes in heart rate. However, because of age-related factors, the heart rate should not be directly used as an index for measuring exercise intensity. Santos reported that the aging process significantly alters the mean heart rate, which decreases with advancing age [9]. Therefore, the mean heart rates of young adults and elderly people at rest differ. In the present study, we used the average maximum heart rate ratio (AMHRR) [10,11], which can reduce the effect of age on the resting heart rate and maximum heart rate in response to exercise, and hypothesized that the AMHRR would facilitate the comparison of EEG and electromyography (EMG) readings between elderly people and young adults at the same exercise intensity.

We measured cardiac, cerebral, and muscular activity levels in elderly people and young adults in response to cycling exercise and investigated the differences between physiological signals obtained from four study groups: regularly exercising elderly people, occasionally exercising elderly people, regularly exercising young people, and occasionally exercising young people. In general, under a constant cycling period and intensity, regularly exercising young adults could achieve higher exercise efficiency with lower brain activation compared with the other participants. However, occasionally exercising young adults and elderly people may need to recruit more muscle units and increase the activation of the motor cortex during cycling compared with regularly exercising young adults. We hypothesized that physiological signal patterns would be similar between the occasionally exercising young adults and elderly people. We also anticipated that as age increases, the significant differences of physiological signals between occasional and regular exercisers may be more obvious in elderly adults than in young adults.

## 2. Materials and Methods

### 2.1. Participants and Data Acquisition

**Figure 1:**
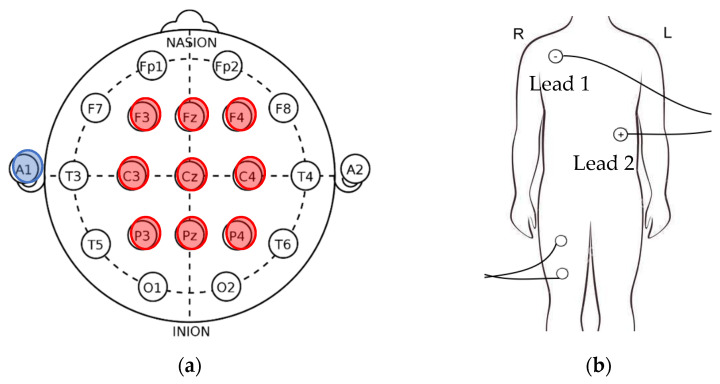
Locations of electrodes for EMG, ECG, and EEG. (**a**) location of electrodes for EEG (**b**) location of electrodes for ECG and EMG.

This study included 36 healthy young adults (18 men and 18 women aged 22.39 ± 3.56 years) and 35 elderly people (20 men and 15 women aged 64.65 ± 2.21 years) as participants. The elderly participants as well as the young participants were subdivided into 2 groups according to the time spent on exercise per week; specifically, participants who exercised for a total time of more than 3 h every week were considered as regularly exercising individuals, and those exercised for a total time of less than 3 h every week were regarded as occasionally exercising individuals [12]. All participants provided informed consent after receiving a detailed explanation of the purpose and potential benefits, and risks involved in the study. This study was conducted according to the guidelines of the Declaration of Helsinki and approved by the Institutional Review Board of National Yang Ming Chiao Tung University (YM106115E-1, 7 March 2019). Moreover, all participants were confirmed by physicians that their body mass index (BMI) was not overweight and without any lower limb or pelvic injuries, and had no brain-related diseases such as stroke, epilepsy, neurodegenerative diseases, orthopedic, or cardiovascular diseases. ECG, EMG, and EEG signals were recorded using V-AMP (Brain Products GmbH, Munich, Germany) with a 1 kHz sampling rate. The EEG channels included 10 wired wet electrodes, namely F3, F4, Fz, C3, C4, Cz, P3, P4, Pz, and A1, and were used according to the international 10/20 system (Figure 1a) [13]. The ground electrode was positioned at FPz. The EEG impedance level was maintained at <20 kΩ during the recording. The A1 channel was used as the reference for all electrodes.

Electrocardiography (ECG) signals were recorded using 2 bipolar lead electrodes. The lead 1 (negative) electrode was situated below the right clavicle, on the mid-clavicular line within the rib cage frame; the lead 2 (positive) electrode was placed on the lower left abdomen, also within the rib cage frame. The surface EMG (sEMG) electrodes were placed on the quadriceps muscle (Figure 1b).

### 2.2. Experimental Protocol

We conducted an experiment to record EEG, ECG, and EMG signals while the participants performed the cycling exercise. Each of the participants sat on an electronically braked cycle ergometer in the upright position, with electrodes attached to their body. The study involved a pretest session and an experimental session. The pretest session involved 10 40 s stages of increasing workload with 20 s of rest between stages. For every participant, the workload ranged from 1 to 10. After the pretest session, the participants took a 5 min rest. The root mean square (RMS) amplitudes of EMG signals recorded for each stage were calculated, and the maximum RMS amplitude was considered the subject-specific maximum workload. For a participant, a workload corresponding to 40% of the maximum RMS amplitude was defined as the suitable workload for this participant. For safety, we assigned lighter exercise loads to the elderly participants to avoid injury or muscle damage due to over-load, considering the effects of declining physiological function with aging. Hence, the pretest session was considered to be excessively strenuous for the elderly participants, their suitable workload was set to 3.

**Figure 2:**
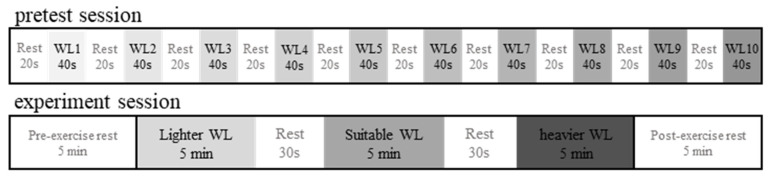
Overall experimental protocol.

In the experimental session, the participants were asked to ride the bicycle in 3 5-min exercise stages, resting for 30 s between stages. These 3 stages corresponded to relatively light, suitable, and relatively heavy workloads. EEG, ECG, and EMG signals were recorded simultaneously while the participants performed the exercise. Signals were also recorded for 5 min before the exercise (pre-exercise period) and for another 5 min after the exercise (post-exercise period). In this study, we required subjects to minimize their head and upper body movement as much as possible during the experiment. The participants were also asked not to move during the resting period. Figure 2 illustrates the overall experimental protocol.

### 2.3. Data Analysis

#### 2.3.1. ECG Analysis

ECG signals were detrended to remove low-frequency shifts, and the peak-to-peak R waves were identified to calculate RR intervals. The RR interval is the time elapsed between 2 successive R waves of the QRS signal on the ECG. We further used the AMHRR to monitor the status of each participant during the experiment [14]. The AMHRR can be defined as follows:

(1) $$\mathrm{AMHRR}=\frac{\mathrm{averaged}\;\mathrm{heart}\;\mathrm{rate}\;\mathrm{in}\;\mathrm{each}\;\mathrm{stage}-\mathrm{RHR}}{\mathrm{predicted}\;\mathrm{maximal}\;\mathrm{heart}\;\mathrm{rate}\left(220-\mathrm{age}-\mathrm{RHR}\right)}\times 100\%$$

where RHR is the average heart rate during rest [10,11].

#### 2.3.2. EEG Analysis

For each participant, EEG signals recorded during the 5 min rest and during the exercise sessions were subjected to band-pass filtering between 1 and 45 Hz. Although participants were advised not to blink their eyes, clench their teeth, tense their muscles, or move their heads, these activities occasionally occurred and introduced artifacts into the EEG data. All signals with these artifacts were discarded during offline data processing. We further applied a moving average to the remaining signals for artifact suppression. Subsequently, each signal was divided into non-overlapping 1 min segments and then subjected to the wavelet transform [15].

The wavelet transform is based on small wavelets with a limited duration. The wavelet transform of a continuous-time signal *x*(*t*) can be defined as follows:

(2) $$\mathrm{WT}\left(a,b\right)=\int_{-\infty }^{\infty }x\left(t\right)\psi_{\left(a,b\right)}\left(t\right)dt$$

where

(3) $$\psi_{\left(a,b\right)}\left(t\right)=\frac{1}{\sqrt{\left|a\right|}}\psi \left(\frac{t-b}{a}\right)$$

is called the mother wavelet. The notations *a* and *b* denote the dimensionless frequency scale variable and time-like translation variable, respectively. The Wavelet transform enables the achievement of excellent localization both in the time domain through translations of the mother wavelet and in the scale (frequency) domain through dilations.

**Figure 3:**
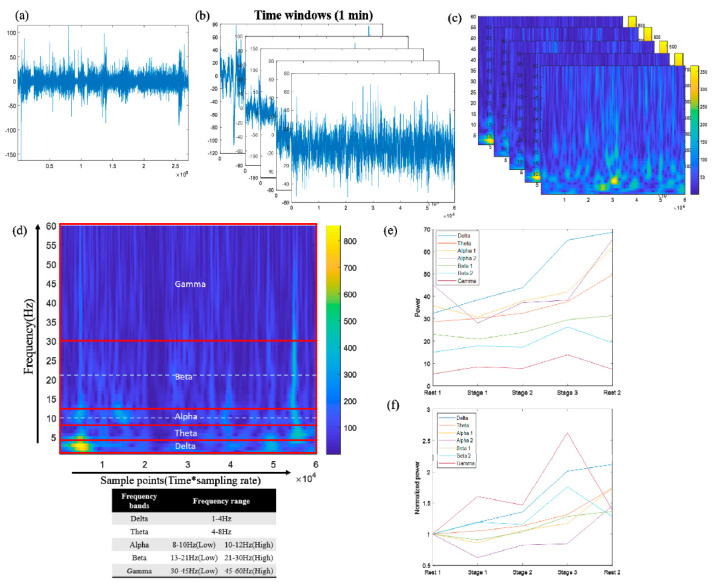
EEG signal analysis procedure. (**a**) Five-minute EEG signals during exercise. (**b**) Five-minute signals divided into 1-min segments. (**c**) Temporal–spectral maps after the application of the Morlet wavelet transform on 1-min segments. (**d**) Temporal–spectral map divided into seven bands: delta, theta, low-alpha, high-alpha, low-beta, high-beta, and gamma bands. (**e**) Average power of each frequency band in each exercise stage. (**f**) Normalized average power of each frequency band in each exercise stage.

In this study, we used the Morlet wavelet [15] to transform each 1 min non-overlapping segment of an EEG signal (Figure 3b) in the 9 channels into temporal-spectral maps (Figure 3c). Each of these maps had 60,000 samples on the horizontal axis and 7 passbands—namely 1–4 (delta), 4–8 (theta), 8–10 (low alpha), 10–12 (high alpha), 13–21 (low beta), 21–30 (high beta), and 31–45 Hz (gamma) Hz—on the vertical axis (Figure 3d). The spectral power levels in each frequency band were averaged to obtain a frequency-averaged temporal power curve, which was again averaged across time to derive a frequency-time-averaged value. Thus, the average power per minute per frequency band was calculated. Each exercise stage was 5 min. Thus, the average power was calculated for 3 different workloads (Figure 3e). Subsequently, to normalize the average power for each exercise stage, this power was divided by the power at rest before exercise, thus yielding the normalized power (Figure 3f).

#### 2.3.3. EMG Analysis

The EMG signals were detrended to remove low-frequency shifts caused by the position fluctuations produced during the cycling exercise. The EMG signals were then subjected to band-stop filtering between 55 and 65 Hz for the removal of noise effects. After preprocessing, the signals were divided into 5 s segments (5000 sample points). RMS is usually used to predict muscle activity. Generally, a higher RMS value means higher muscle activity. RMS can be derived as follows:

(4) $$\mathrm{RMS}=\sqrt{\frac{1}{N}\overset{N}{\underset{n=1}{\sum }}x_{n}^{2}}$$

where $x_{n}^{2}$ represents the EMG signal and *N* represents the length of the signal.

#### 2.3.4. Statistical Analysis

**Figure 4:**
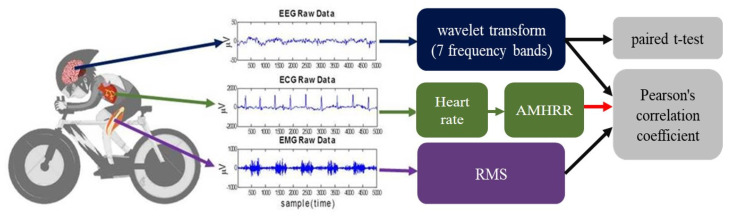
EEG, ECG, and EMG procedures in this study.

Pearson correlation analysis was used to evaluate the linear relationships between normalized power of EEG and the AMHRR or RMS of EMG. The AMHRR was considered an indicator of heart load for the various exercise stages. Thus, we could observe EEG and EMG changes with different exercise loads. In addition, paired-sample *t*-tests were used to examine for significant within-group changes before and after exercise (stage 3 and rest 2) to determine the post-exercise recovery status. In this study, MATLAB R2013b software (Mathworks, Natick, MA, USA) was applied for data analysis. Figure 4 illustrates a summary of the analysis procedures of EEG, ECG and EMG used in this study.

## 3. Results

### 3.1. Changes in Heart Rate and AMHRR with Exercise Stages

**Figure 5:**
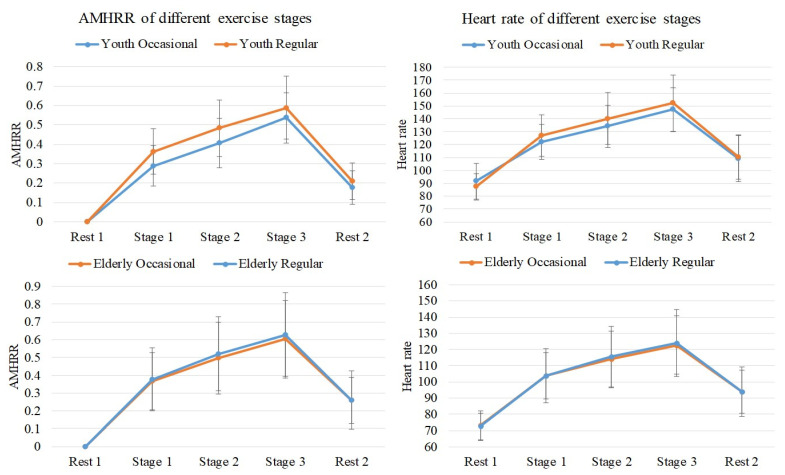
ECG analysis results for mean heart rate and AMHRR.

The mean heart rate and the AMHRR of the young and elderly participants during the different exercise stages are presented in Table 1 and Table 2. Figure 5 illustrates the ECG analysis results for mean heart rate and AMHRR.The results revealed that in all groups, the heart rate and the AMHRR increased gradually with each exercise stage. The heart rates of the elderly participants were lower than those of the young participants. However, the AMHRR values of the elderly participants were not significantly different from those of the young participants, indicating that the cardiac load conditions of both the young and elderly participants were similar. The AMHRR was derived by normalizing the heart rate and excluding the effects of basal heart rate and age. Therefore, the AMHRR was suitable for observing the physiological state of the heart. We used Pearson correlation coefficient analysis to estimate the association between normalized EEG power and AMHRR per minute.

### 3.2. Changes in EEG during Exercise in Young Participants with and without Exercise Habits

We used Pearson correlation analysis to estimate the correlation between normalized EEG power and the AMHRR. Table 3 presents a summary of the regression coefficients of normalized EEG power and the AMHRR for all frequency bands. According to this table, a moderately strong correlation was observed, with the normalized coefficient ranging from 0.4 to 0.6. The results demonstrated that changes in EEG at the most frequency bands at C3, C4, and Cz were significantly and positively correlated with the AMHRR in both the young and elderly participants (*p* < 0.001). Moreover, the effect of exercise on EEG was mainly observed in the alpha band.

According to Table 3, we could also observe the effect of exercise habits on normalized EEG power in the young participants. The correlation between normalized EEG power at C3 and the AMHRR was higher in young participants who exercised regularly, and the correlation between normalized EEG power at C4 and the AMHRR was higher in young participants who exercised occasionally.

### 3.3. Changes in EEG during Exercise in Elderly Participants

**Figure 6:**
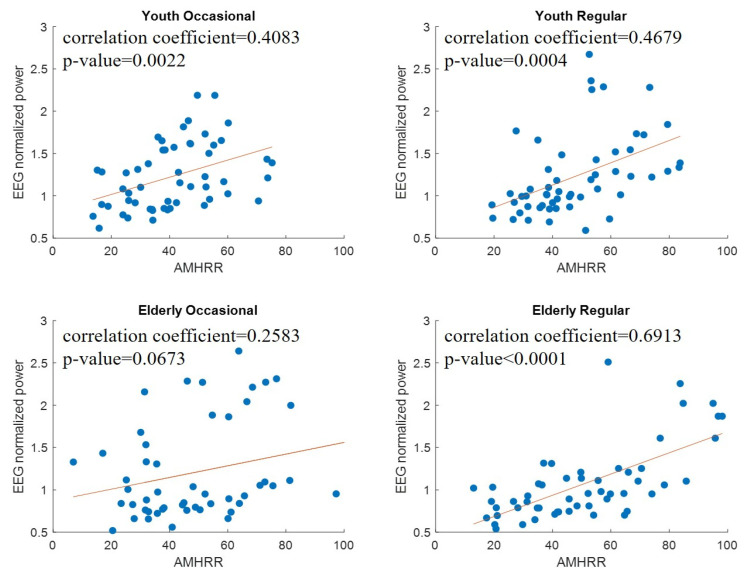
Scatter plots of correlation between normalized EEG power and AMHRR in low-alpha band (C3).

As presented in Table 3, the regression coefficients revealed a moderate or high correlation between normalized EEG power and the AMHRR in the elderly participants who exercised regularly. However, the correlation observed for the elderly participants who exercised occasionally was low and nonsignificant. Combining the results for elderly participants and young participants revealed that maintaining adequate exercise habits was more imperative for older adults than for younger adults. As illustrated in Figure 6, the elderly participants who exercised regularly demonstrated consistent EEG power changes. As the AMHRR increased, the normalized EEG power also increased. By contrast, no clear trend was observed for the elderly participants who exercised occasionally. The changes in EEG power were more dispersed. These results indicated that adequate exercise habits may lead to more stable brain wave changes in elderly people during exercise.

### 3.4. Paired t-Test Results Observed during and after Exercise

**Figure 7:**
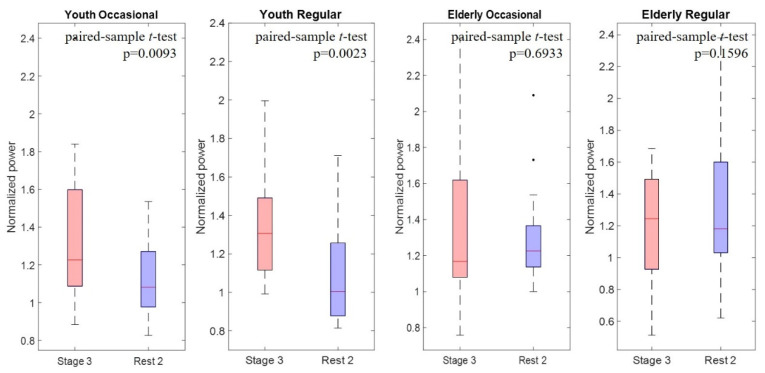
Histogram of EEG normalized power in low-beta (C3) band during exercise stage 3 and post-exercise rest.

Figure 7 displays the normalized power values and statistical analysis results observed at C3 (low beta) at stage 3 and during post-exercise rest. Accordingly, the normalized power value during post-exercise rest would decrease to 1 if the power value during the pre- and post-exercise rest periods were identical. According to the plots in Figure 7, we observed the recovery speed of EEG power after exercise. The results revealed a significant difference in the change in normalized power between the exercise stage and post-exercise rest state in the young participants, regardless of their exercise habits. By contrast, in the elderly participants, the difference in the change in normalized power between stage 3 and post-exercise rest states was nonsignificant. Table 4 presents a summary of the results of the paired *t*-test for normalized EEG power in stage 3 and in the post-exercise rest state. In particular, the difference between the young and elderly participants was clearly observed in the beta band. The young participants recovered faster after exercise; therefore, a significant difference in the change in normalized power was observed. By contrast, the elderly participants recovered more slowly after exercise; hence, the difference in the change in normalized power was nonsignificant.

Overall, the alpha and beta bands could reflect changes in brain wave power during and after exercise. The alpha band can be used to observe changes in brain wave power during exercise, and the beta band can be used to observe recovery in rest states after exercise. However, further understanding of the effect of age and exercise habits on EEG changes is warranted.

### 3.5. Relationship between EMG RMS and AMHRR for the Four Test Groups

**Figure 8:**
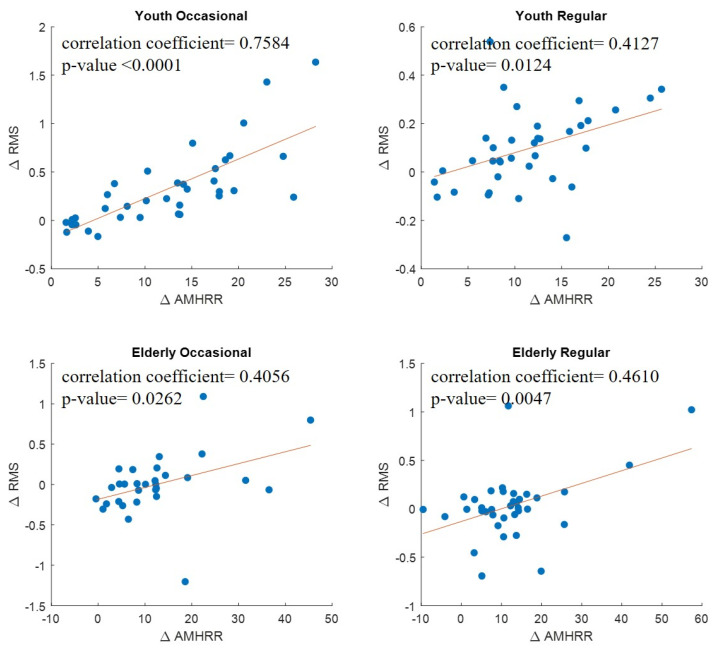
Linear regression between △EMG RMS and △AMHRR.

Figure 8 displays the results of the linear regression on the differences in EMG RMS between stages 2 and 1 (i.e., ΔEMG RMS) and the difference in AMHRR between stages 2 and 1 (i.e., ΔAMHRR). Because of the extensive individual differences in EMG RMS values and the location of the EMG bipolar electrodes, we normalized the EMG RMS values; that is, we divided the RMS values for stages 2 and 3 by those for stage 1. This can be used to observe the increase in ΔEMG RMS with exercise load. The results revealed a more significant trend of increasing ΔEMG RMS with ΔAMHRR in the young participants than in the elderly participants. Additionally, the regression coefficients for the young participants who exercised occasionally were higher than those for the young participants who exercised regularly. However, for the elderly participants, a low correlation was observed between the ΔEMG RMS values and ΔAMHRR, regardless of their exercise habits, and their ΔEMG RMS values were more clustered. This low correlation may be because in this study, the elderly participants were assigned a fixed cycling load that was lower than those assigned to the young participants.

## 4. Discussion

This study used ECG, EMG, and EEG to explore changes in physiological signals transmitted during cycling exercise in young and elderly participants with different exercise habits. We assigned lighter exercise loads to the elderly participants to avoid muscle damage or injury from overload, considering the effects of declining physiological function with aging. According to previous research, exercise intensity (workload) is reflected in the response of many physiological processes, including heart rate [16]. Therefore, we defined the exercise load according to the AMHRR and further observed changes in EEG and EMG with gradually increasing exercise loads.

### 4.1. Spectral Power of EEG Increases with AMHRR during Exercise

We observed that during exercise, the normalized power of each frequency band of the EEG signal was positively and linearly correlated with the AMHRR. We also determined that an increase in normalized EEG power was consistent with an increase in AMHRR. This consistency was observed in most EEG frequency bands, including the delta, theta, low-alpha, high-alpha, low-beta, and high-beta bands. Furthermore, these phenomena were more evident in the low-alpha, high-alpha, low-beta, and high-beta frequency bands. Earlier research reported that cortical activity increased with fatigue during exercise in order to maintain a constant physical output [1]. Schillings et al. also reported that the energy loss associated with fatigue during exercise may cause increased brain activation in the motor cortex [17].

Previous studies determined that during exercise, EEG cortical activation was most affected in the alpha and beta frequency bands [18,19,20,21]. Therefore, most experiments and literature reviews on the effects of exercise on EEG cortical activity were limited to these two frequency bands. Several previous studies involving ergometer cycling revealed that incremental graded exercise tests resulted in increased alpha power in the central and parietal regions as well as increased EEG current density in the primary motor region. Bailey et al. showed an increase in alpha and beta power after sustained intensity bicycle ergometer exercise with a progressively increasing workload [3]. Lin et al. reported increased EEG power in the alpha and beta bands in the frontal and central areas during high-resistance pedaling exercise [22]. They further proposed that the fatigue situation would be accompanied by an increase in α and β power. However, increased EEG beta activity may be associated with attentional demands and higher levels of arousal. Other studies demonstrated that the effect of exercise on EEG cortical activity was not limited to the alpha and beta bands [3,5]. Our results demonstrated that the alpha band was more suitable for observing changes in brain activation during exercise. However, the beta band was more appropriate for determining the differences between brain activation observed during exercise and that observed during post-exercise rest.

### 4.2. Young People Who Exercise Regularly Have a More Coordinated Use of Their Dominant Leg

Our results reveal that the EEG differences between young participants who exercised regularly and those who exercised occasionally were in the activation of motor cortical areas in the left and right hemispheres (i.e., C3 and C4). A higher correlation was observed between normalized power changes at C3 and exercise load in the young participants who exercised regularly. However, the normalized power at C3 and that at C4 in the young participants who exercised occasionally were moderately correlated. The concept of limb dominance was based on the fact that the two hemispheres of the brain function differently and tend toward activities that use one limb under voluntary control [23]. Bhise et al. observed that when for an inherently manipulative task, most participants used the dominant leg [24]. Young people who exercise regularly have greater coordination in the use of the dominant leg, meaning that they require only the dominant leg to complete the exercise. However, young people who exercise occasionally must use both legs to compensate for the deficiency of the dominant leg [25,26,27]. The RMS of EMG signals is often used as a concise quantitative indicator of muscle activity; we found that the young participants who exercised occasionally had significantly higher EMG RMS values than did those who exercised regularly. Our results indicate that the dominant legs of young people who exercise occasionally require more force output to perform a given task. However, that the young participants who exercised occasionally had lower EEG activation in the C3 region than did those who exercised regularly.

### 4.3. Regular Exercise in Elderly People Induces Significant Cortical Activation during Exercise

We observed that the highest increase in EEG normalized power occurred when the participants were at their highest AMHRR (exercise workload). This phenomenon was particularly notable in the elderly participants who exercised regularly. The results reveal that the normalized EEG power increased with the AMHRR in the elderly participants who exercised regularly, with the corresponding correlation being moderate to high. The heart rate increases with the delivery of oxygenated blood around the body and into the brain. Muscles require relatively high energy during exercise. Similarly, the brain consumes glucose or other carbohydrates when the body is in motion [28]. Therefore, the brain becomes more active during exercise. This suggests that elderly people who exercise regularly require relatively high exercise performance and muscle strength during exercise, which may induce considerable activation of the cerebral cortex. However, the change in normalized EEG power with respect to exercise load was less consistent in the elderly participants who exercised occasionally. Accordingly, the results reveal no significant correlation between normalized EEG power and the AMHRR. Although this phenomenon could also be observed in the young participants, the results were less pronounced than those observed in the elderly participants. Our results show that the difference in EEG signal changes between the elderly participants who exercised occasionally and those who exercised regularly was more significant than that between the young participants who exercised occasionally and those who exercised regularly. For elderly people, regular exercise can help reduce the functional decline associated with aging.

### 4.4. EEG Recovery after Exercise Is Slower in Elderly People

The paired *t*-test revealed significant beta band activation in the young participants in stage 3 and during post-exercise rest. By contrast, this phenomenon was not observed in the elderly participants. These results indicate that the young participants returned to a resting state more quickly after exercise, whereas the elderly participants required a longer time to recover. Aging affects the post-exercise recovery process. Several studies have revealed a functional decrease in the replenishment of energy supply before and after exercise. Research has presented evidence of differences in acute recovery of physiological parameters after fatiguing exercise between younger and older participants. For similar exercise stimuli, elderly people require a longer recovery period when returning to baseline levels after exercise [29]. Although this study did not reveal a significant difference in exercise recovery between the elderly participants with and without exercise habits, the EEG results demonstrate that the elderly participants who exercised regularly had superior brain regulation of exercise load than did those who exercised occasionally.

However, there are still some limitations in this study. First, the muscle artifacts occurring in the head and neck musculature during cycling exercise may be recorded in EEG signals. In this study, we asked subjects to minimize their head and upper body movement as much as possible during the experiment. Unfortunately, experimental protocols are still sensitive to physiological and non-physiological artifacts, including motion artifacts that may contaminate the EEG recordings. Following the procedure of artifact suppression, we applied a simple cleaning noise method, moving average, to remove the noise caused from motion artifacts in EEG signals. Although these procedures can eliminate motion artifacts but may also decrease the sensitivity in EEG signals. Second, the strength of muscle will decrease with aging, and there exist an individual difference in this aging effect. In this study, we did not take the muscle strength decay of elderly and individual muscle ability into consideration in the experiment setup.

## 5. Conclusions

This study revealed the AMHRR to be a suitable indicator of exercise intensity and that the physiological indicators of ECG and EEG in elderly people are different from those in young people because of aging. We found that the EEG spectral power of elders who exercised regularly revealed the strongest positive correlation with their exercise intensity. The results demonstrate that exercise-induced significant cortical activation in the elderly participants who exercised regularly, and most of the *p*-values are less than 0.001. No significant correlation was observed between spectral power and exercise intensity in the elders who exercised occasionally. The young participants who exercised regularly had greater cardiac and neurobiological efficiency. Therefore, appropriate exercise habits may benefit brain responsiveness and improve the efficiency of cardiac and neurobiological responses to exercise. Our results may provide a new exercise therapy reference for adult groups with different exercise habits, especially for the elders.

## Figures and Tables

**Table 1:** ANOVA results for heart rate in young and elderly participants during different exercise stages. * Indicates *p*-value < 0.05.

		Heart Rate (BPm)			
		Mean	SD	F	Post Hoc Test (*p* < 0.05)
Rest 1	Youth Regular	87.26	10.39	14.97 *	Elderly Regular, Occasional
	Youth Occasional	91.67	13.86		Elderly Regular, Occasional
	Elderly Regular	72.57	8.18		Youth Regular, Occasional
	Elderly Occasional	73.07	9.27		Youth Regular, Occasional
Stage 1	Youth Regular	127.33	16.11	11.65 *	Elderly Regular, Occasional
	Youth Occasional	122.19	13.39		Elderly Regular, Occasional
	Elderly Regular	103.95	16.63		Youth Regular, Occasional
	Elderly Occasional	103.65	14.23		Youth Regular, Occasional
Stage 2	Youth Regular	140.34	19.90	9.35 *	Elderly Regular, Occasional
	Youth Occasional	134.19	16.38		Elderly Regular, Occasional
	Elderly Regular	115.43	19.18		Youth Regular, Occasional
	Elderly Occasional	114.12	17.14		Youth Regular, Occasional
Stage 3	Youth Regular	152.12	21.66	10.81 *	Elderly Regular, Occasional
	Youth Occasional	147.16	17.20		Elderly Regular, Occasional
	Elderly Regular	124.22	20.69		Youth Regular, Occasional
	Elderly Occasional	122.78	18.06		Youth Regular, Occasional
Rest 2	Youth Regular	110.19	16.87	5.65	
	Youth Occasional	109.42	18.10		
	Elderly Regular	94.03	15.45		
	Elderly Occasional	94.20	13.32		

**Table 2:** ANOVA results for AMHRR in young and elderly participants during different exercise stages.

		AMHRR (%)			
		Mean	SD	F	Post Hoc Test (*p* < 0.05)
Rest 1	Youth Regular				
	Youth Occasional				
	Elderly Regular				
	Elderly Occasional				
Stage 1	Youth Regular	36.33	11.79	1.47	
	Youth Occasional	28.29	10.36		
	Elderly Regular	37.78	17.69		
	Elderly Occasional	36.89	16.17		
Stage 2	Youth Regular	48.33	14.58	1.41	
	Youth Occasional	40.72	12.84		
	Elderly Regular	51.98	20.87		
	Elderly Occasional	49.79	20.29		
Stage 3	Youth Regular	58.96	16.26	0.71	
	Youth Occasional	53.69	12.83		
	Elderly Regular	62.54	24.02		
	Elderly Occasional	60.56	21.28		
Rest 2	Youth Regular	20.95	9.60	1.96	
	Youth Occasional	17.59	8.66		
	Elderly Regular	26.08	16.53		
	Elderly Occasional	25.86	13.06		

**Table 3:** Regression coefficients for the correlation between normalized EEG power and AMHRR during exercise. Boldface values represent moderate positive correlation between normalized EEG power and the AMHRR (correlation coefficient > 0.4).

		Delta	Theta	L-Alpha	H-Alpha	L-Beta	H-Beta	Gamma
C3	Youth Occasional	0.1787 (0.1959)	0.2137 (0.1207)	0.4083 (0.0022)	0.4699 (0.0003)	0.3848 (0.0041)	0.2407 (0.0796)	0.1770 (0.2004)
	Youth Regular	0.4426 (0.0008)	0.4215 (0.0015)	0.4679 (0.0004)	0.4493 (0.0007)	0.3831 (0.0042)	0.2130 (0.1220)	0.1404 (0.3113)
	Elderly Occasional	0.3759 (0.0066)	0.2405 (0.0892)	0.2583 (0.0673)	0.2672 (0.0580)	0.2463 (0.0815)	0.2088 (0.1414)	0.2381 (0.0925)
	Elderly Regular	0.7037 (<0.0001)	0.6519 (<0.0001)	0.6913 (<0.0001)	0.6441 (<0.0001)	0.5516 (<0.0001)	0.5376 (<0.0001)	0.5284 (<0.0001)
C4	Youth Occasional	0.2498 (0.0685)	0.2907 (0.0329)	0.4961 (0.0001)	0.5641 (<0.0001)	0.4637 (0.0004)	0.3110 (0.0221)	0.2288 (0.0961)
	Youth Regular	0.2103 (0.1268)	0.2221 (0.1065)	0.3645 (0.0067)	0.3643 (0.0068)	0.2607 (0.0569)	0.0716 (0.6068)	−0.0280 (0.8406)
	Elderly Occasional	0.3585 (0.0098)	0.2222 (0.1170)	0.2012 (0.1569)	0.2087 (0.1417)	0.2184 (0.1237)	0.1866 (0.1899)	0.2366 (0.0947)
	Elderly Regular	0.6107 (<0.0001)	0.6164 (<0.0001)	0.6644 (<0.0001)	0.6070 (<0.0001)	0.3623 (0.0071)	0.2948 (0.0305)	0.2788 (0.0412)
Cz	Youth Occasional	0.1729 (0.2112)	0.2076 (0.1319)	0.3869 (0.0038)	0.4639 (0.0004)	0.3673 (0.0063)	0.2385 (0.0824)	0.1706 (0.2176)
	Youth Regular	0.2310 (0.0929)	0.2845 (0.0371)	0.4181 (0.0017)	0.4113 (0.0020)	0.3397 (0.0120)	0.1716 (0.2146)	0.0650 (0.6404)
	Elderly Occasional	0.3904 (0.0046)	0.2652 (0.0600)	0.2624 (0.0629)	0.2700 (0.0553)	0.2668 (0.0584)	0.2342 (0.0981)	0.2798 (0.0468)
	Elderly Regular	0.4380 (0.0009)	0.4607 (0.0005)	0.5498 (<0.0001)	0.5505 (<0.0001)	0.4959 (0.0001)	0.5027 (0.0001)	0.4986 (0.0001)

**Table 4:** *p* values for paired-sample *t*-tests of normalized EEG power during and after exercise.

		Delta	Theta	L-Alpha	H-Alpha	L-Beta	H-Beta	Gamma
C3	Youth Occasional	*p* = 0.0861	*p* = 0.0872	*p* = 0.1032	*p* = 0.0545	*p* = 0.0093	*p* = 0.0272	*p* = 0.0064
	Youth Regular	*p* < 0.001	*p* = 0.0011	*p* = 0.6801	*p* = 0.5305	*p* = 0.0023	*p* = 0.0011	*p* < 0.001
	Elderly Occasional	*p* = 0.0122	*p* = 0.0621	*p* = 0.9527	*p* = 0.9249	*p* = 0.6933	*p* = 0.1791	*p* = 0.0027
	Elderly Regular	*p* = 0.1019	*p* = 0.6662	*p* = 0.0180	*p* = 0.0324	*p* = 0.1596	*p* = 0.3657	*p* = 0.3446
C4	Youth Occasional	*p* = 0.0658	*p* = 0.0499	*p* = 0.0738	*p* = 0.0348	*p* = 0.0049	*p* = 0.0151	*p* = 0.0034
	Youth Regular	*p* < 0.001	*p* = 0.0022	*p* = 0.4626	*p* = 0.4761	*p* = 0.0021	*p* < 0.001	*p* < 0.001
	Elderly Occasional	*p* = 0.0240	*p* = 0.0543	*p* = 0.7284	*p* = 0.8185	*p* = 0.5308	*p* = 0.1379	*p* = 0.0017
	Elderly Regular	*p* = 0.0122	*p* = 0.4295	*p* = 0.0197	*p* = 0.0569	*p* = 0.5484	*p* = 0.9590	*p* = 0.1634
Cz	Youth Occasional	*p* = 0.0808	*p* = 0.0902	*p* = 0.1752	*p* = 0.0640	*p* = 0.0106	*p* = 0.0252	*p* = 0.0045
	Youth Regular	*p* = 0.0015	*p* = 0.0211	*p* = 0.9572	*p* = 0.8219	*p* = 0.0109	*p* = 0.0037	*p* < 0.001
	Elderly Occasional	*p* = 0.0254	*p* = 0.0952	*p* = 0.8433	*p* = 0.8706	*p* = 0.9117	*p* = 0.4567	*p* = 0.0040
	Elderly Regular	*p* = 0.1706	*p* = 0.3402	*p* = 0.6988	*p* = 0.6412	*p* = 0.7912	*p* = 0.6926	*p* = 0.0321

## Data Availability

The data are not publicly available due to the privacy concern raised by our IRB.
